# First description of underwater acoustic diversity in three temperate ponds

**DOI:** 10.7717/peerj.1393

**Published:** 2015-11-05

**Authors:** Camille Desjonquères, Fanny Rybak, Marion Depraetere, Amandine Gasc, Isabelle Le Viol, Sandrine Pavoine, Jérôme Sueur

**Affiliations:** 1 Institut de Systématique, Évolution, Biodiversité, ISYEB—UMR 7205—CNRS, MNHN, UPMC, EPHE, Muséum national d’Histoire naturelle, Sorbonne Universités, Rue Buffon, Paris, France; 2 Université Paris-Sud, Neuroscience Paris-Saclay Institute, UMR 9197, Orsay, France; 3 Ecologie et Gestion de la Biodiversité, Centre d’Ecologie et des Sciences de la Conservation (CESCO UMR7204), Sorbonne Universités, MNHN, CNRS, UPMC, Paris, France; 4 Centre d’Ecologie et des Sciences de la Conservation (CESCO UMR7204), Museum national d’Histoire naturelle, Paris, France; 5 Mathematical Ecology Research Group, Department of Zoology, University of Oxford, Oxford, United Kingdom

**Keywords:** Ponds, Sound, Monitoring, Acoustic diversity indices

## Abstract

The past decade has produced an increased ecological interest in sonic environments, or soundscapes. However, despite this rise in interest and technological improvements that allow for long-term acoustic surveys in various environments, some habitats’ soundscapes remain to be explored. Ponds, and more generally freshwater habitats, are one of these acoustically unexplored environments. Here we undertook the first long term acoustic monitoring of three temperate ponds in France. By aural and visual inspection of a selection of recordings, we identified 48 different sound types, and according to the rarefaction curves we calculated, more sound types are likely present in one of the three ponds. The richness of sound types varied significantly across ponds. Surprisingly, there was no pond-to-pond daily consistency of sound type richness variation; each pond had its own daily patterns of activity. We also explored the possibility of using six acoustic diversity indices to conduct rapid biodiversity assessments in temperate ponds. We found that all indices were sensitive to the background noise as estimated through correlations with the signal-to-noise ratio (SNR). However, we determined that the *AR* index could be a good candidate to measure acoustic diversities using partial correlations with the SNR as a control variable. Yet, research is still required to automatically compute the SNR in order to apply this index on a large data set of recordings. The results showed that these three temperate ponds host a high level of acoustic diversity in which the soundscapes were variable not only between but also within the ponds. The sources producing this diversity of sounds and the drivers of difference in daily song type richness variation both require further investigation. Such research would yield insights into the biodiversity and ecology of temperate ponds.

## Introduction

Over the past 15 years, scientists and land managers have started to draw attention to the importance of ponds in terms of patrimonial, recreational, decorative, agricultural, ecological and environmental interests (Oertli et al., 2009). These water bodies are occupied by a large diversity of organisms harbouring an important number of endemic vertebrate and invertebrate species, some of which are highly threatened (Céréghino et al., 2012). Natural processes such as sedimentation or seasonal changes modify ponds and their environmental parameters, they are thus most of the time bound to be temporary (Wood, Greenwood & Agnew, 2003). Ponds are experiencing an unprecedented and severe degradation due to anthropological causes such as drainage, ancient custom abandon (e.g., forest or agricultural ponds), urbanisation or agriculture intensification (Wood, Greenwood & Agnew, 2003). This degradation can take several forms such as habitat fragmentation, reduction, quality alteration (e.g., pollution or desiccation) or even complete habitat loss. Hence, depending on the area, 40–90% of European ponds have disappeared during the twentieth century (United Kingdom: Wood, Greenwood & Agnew, 2003, Europe: Hull, 1997). Combining a unique diversity and a high level of threat, many ponds can be therefore considered as habitats of high concern for biodiversity conservation, in particular under temperate climates (Verberk et al., 2006).

Plant and animal diversity of ponds has been well studied, however an original facet of biodiversity, the acoustic diversity due to the acoustic signals produced by animals, has been totally neglected. The description and analysis of acoustic diversity was proven to be a valuable approach of diversity assessment combining results in animal behaviour and ecology, in particular to estimate the space and time distribution of species (Towsey, Parsons & Sueur, 2014). The acoustics of freshwater ecosystems have rarely been investigated. Yet they are inhabited by many species belonging to taxa for which terrestrial as well as some aquatic members are known to produce sounds. In ponds, a high diversity of amphibians generates sound underwater during breeding (Duguet & Melki, 2003). Sound production in freshwater arthropods is also quite common but has not been given as much attention. A few crayfish species have been proven to produce sound (Sandeman & Wilkens, 1982; Favaro et al., 2011). Moreover insect stridulation probably makes up most of the acoustic diversity in ponds (Aiken, 1985). A comprehensive review of sound-producing aquatic insects lists 15 families belonging to four orders, namely Trichoptera, Odonata, Coleoptera and the order with the largest number of sound-producing species, Heteroptera (Aiken, 1985). The underwater insect signals cover a wide frequency bandwidth, ranging from 200 Hz for the Coleoptera imago *Acilius sulcatus* (Leston, Pringle & White, 1965) up to 100 kHz for Hydropsychidae larvae (Trichoptera, Silver & Halls, 1980).

Sounds produced by animals can be considered as interacting items belonging to a high-level ecological organization such as a community or a landscape (Farina, 2014). Ecoacoustics, a newly formed discipline, aims at considering animal sound as a material for ecology and biodiversity monitoring (Sueur & Farina, 2015). In this global approach, sound is mainly considered as a tool to infer ecological information. In practice, recordings do not focus on a single singing species but on the overall acoustic output emanating from a community or a landscape. The analysis of these sounds aims to assess and characterize general features of the structure and the diversity of either an acoustic community, defined as an assemblage of species that share a similar acoustic space (Gasc et al., 2013) or a soundscape, defined as ‘the collection of biological, geophysical and anthropogenic sounds that emanate from a landscape’ (Pijanowski et al., 2011). The global approach is mainly based on the assumption that part of biodiversity can be reflected by acoustic diversity. Numerous acoustic diversity indices have been proposed to measure the acoustic community diversity or soundscape composition (Sueur et al., 2014). All these indices forego species identification and produce relative values that aim at quantifying a feature of the community or the soundscape, like the energy, the complexity or the relative importance of the biophony. The indices have been first tested in terrestrial environments (Sueur et al., 2008; Pieretti, Farina & Morri, 2011; Gage & Axel, 2014; Towsey et al., 2014) and then in marine habitats (Parks, Miksis-Olds & Denes, 2014). These first trials revealed mixed results suggesting the importance of background noise in the reliability of indices (Gasc et al., 2015).

No passive acoustic monitoring study considering all sources of acoustic productions had been conducted in freshwater ponds. We thus explored for the first time the acoustic diversity of three temperate ponds in three different habitats. We tested the following hypothesis: (i) the acoustic diversity differs in richness and composition between the three ponds, (ii) the richness of acoustic diversity varies along day and night revealing ecological cycles, and (iii) acoustic indices can represent the richness of acoustic diversity of each pond detected by human-hearing.

## Material and Methods

### Study area and recordings

Three ponds were monitored in the Parc Naturel Régional de la Haute Vallée de Chevreuse (PNR), a protected area located 40 km south-west of Paris, France. The ponds were located in three different environments differing by the density of the surrounding vegetation: closed forest habitat (pond 1, 48°34.523′N, 1°53.341′E), semi-closed habitat (pond 2, 48°40.772′N, 1°55.840′E) and open field habitat (pond 3, 48°40.560′N, 1°55.865′E). The main characteristics of the three ponds are summarised in Table S1.

Each pond was monitored with an autonomous recording platform including four units: (1) a hydrophone Reson TC4033 (flat frequency response between 20 Hz and 40 kHz) with a 10 m cable, (2) a charge pre-amplifier Avisoft UltraSoundGate with a frequency high-pass filter at 100 Hz and a gain of +20 dB, (3) a digital audio field recorder SM1 (Wildlife Acoustics, Inc., 2015) with a built-in Texas-Instrument anti-alias filter, (4) a 12 V battery connected to the audio recorder and charged with a solar panel. A four-point linear transect was defined to cross each pond to maximize representation of heterogeneous patches of vegetation. Recordings were achieved on each of the four points of this transect to collect spatial heterogeneity within the ponds. A single recording platform was available for each pond. The hydrophone position was changed every three days according to a sampling rotation with a set up allowing the hydrophone to move without any intrusion in the ponds. The hydrophone was placed 10 cm below the water surface to reduce heterogeneity of sound propagation that is depth-dependent in shallow water (Forrest, Miller & Zagar, 1993). Rainfall data were collected from a local meteorological station (Météo France, 2015).

Each recorder was programmed to record during one minute every fifteen minutes between the 23rd of June and the 15th of September 2010 (84 days) when the activity and abundance of macro-invertebrate species are known to be maximal (Gascón, Boix & Sala, 2009). The sampling design (3 ponds × 96 recording time slots × 84 days) resulted in 24,192 files, among which 7,873 were missing due to technical issues (file corruption, material theft and dysfunction). The final number of files obtained was 16,319. These recordings were sampled at 44.1 kHz with a 16 bits digitization. The files were saved in the lossless compressed format .wac and then transformed into the format .wav with the software WAC to WAV Converter Utility version 1.1 (Wildlife Acoustics, Inc., 2015).

### Aural and visual classification of sounds: detection of sound types

Visual identification was conducted on a sub-sample of the initial sample of 16 319 files. Five complete recording days for each sampling point in the three ponds were randomly selected avoiding rainy days. For each selected day, six recording times were defined (00:00, 04:00, 08:00, 12:00, 16:00 and 20:00) resulting in 360 recordings (3 ponds × 4 sampling points × 5 days × 6 recordings per day). These 360 recordings can be considered as samples. 28 recordings, spread across the 3 ponds, had to be withdrawn from this sub-sample due to technical problems with the recorders. Due to the lack of a sound bank for most freshwater species, sounds could not be identified at a species level. Therefore sound types, instead of species-specific sounds, were identified and classified based on aural and visual inspections. Aural inspection was achieved using circumaural headphones and by listening to the files as many times as necessary. This aural inspection was accompanied with the sound visualisation of oscillograms and spectrograms (Window length: 256 samples, frame overlap: 0%, window type: Hanning) with the software Audacity (http://audacity.sourceforge.net/). The classification of sound types was based on similarity in amplitude and dominant frequency contours and achieved only by MD to avoid any bias due to the experimenter. This identification of sound types was summarized by two variables for each recordings: the richness of sound types per recording (hereafter referred to as richness) which is the number of different sound types in a recording and the abundance of sound types per recording (hereafter referred to as abundance) which is the total number of sound types detected in a recording.

### Signal to noise ratio (SNR)

The signal-to-noise ratio (SNR) of each recording was estimated by computing the ratio between the amplitude of one second of signal (extract lasting one second and containing an identified sound type) and the amplitude of one second of noise (e.g., one second of recording without any signal) in each file as follows:

\begin{eqnarray*} \mathrm{SNR}=(A s/A n)^{2}, \end{eqnarray*}

with *As* and *An* the root mean square (RMS) of signal and noise sections respectively. dB values were obtained by computing:

\begin{eqnarray*} {\mathrm{SNR}}_{\mathrm{dB}}=20\ast {\log }_{10}\mathrm{SNR}. \end{eqnarray*}

The SNR of recordings in which no signal could be found was set to one leading to a SNR_dB_ of zero.

### Acoustic analysis

Several acoustic indices have been developed recently to assess the acoustic diversity of a community or a landscape (Sueur et al., 2014). Six acoustic indices were chosen to parametrize the files that were aurally and visually inspected. These indices were (1) the temporal entropy *Ht* that computes the Shannon evenness of the amplitude envelope (Sueur et al., 2008), (2) the spectral entropy *Hf* that computes the Shannon evenness of the mean frequency spectrum (Sueur et al., 2008), (3) the envelope energy *M* that returns the median of the amplitude envelope (Depraetere et al., 2012), (4) the acoustic richness *AR* which is a ranked index based on the multiplication of *Ht* and *M* (Depraetere et al., 2012), (5) the number of major peaks of the mean frequency spectrum *NP* (Gasc et al., 2013), and (6) the Acoustic Complexity Index *ACI* which calculates the complexity of the spectrogram, i.e., of the short-term Fourier transform (Pieretti, Farina & Morri, 2011). More details regarding these indices can be found in Sueur et al. (2014). All spectral data were obtained with a short-term Fourier transform with a 512 samples non-overlapping Hamming window. To obtain *Ht*, the absolute amplitude envelope was computed. For *NP* the parameters used were 1/50 for the amplitude slope threshold and 200 Hz for the frequency threshold. The time step for the *ACI* was 30 s and the frequency bins were 86 Hz. All acoustic analyses were achieved with the package seewave (Sueur, Aubin & Simonis, 2008) of the R statistical environment (R Core Team, 2015).

### Statistical analysis

Richness in sound types were compared between ponds with sample-based rarefaction curves (Simberloff, 1972) obtained with the R package vegan version 2.2-1 (Oksanen et al., 2013).

A Correspondence Analysis (CA) was computed to characterize the community at each recording point along the transect and the way the communities were ordinated considering their sound type composition. Each recording point was considered as a single observation and the presence/absence data for each sound type was used as a variable.

Differences in SNR between ponds were first assessed with an analysis of variance (ANOVA) but the assumptions of normality and homoscedasticity of the model residuals were not met. A Kruskal–Wallis non-parametric test was therefore used followed by pairwise comparisons using Wilcoxon rank sum test with a Bonferroni adjustment.

To investigate the effect of time in the ponds on sound type richness per recording, we used a Generalized Linear Mixed Model (GLMM; Baayen, 2008) with a Poisson error structure and log link function (Mc Cullagh & Nelder, 1989). To examine the daily cyclic effects of time on the richness, we transformed time into a circular variable and included its sine and cosine into the model (Cox, 2006). Since the effect of time is likely to vary among ponds if they host different species, we included the interaction between pond and the sine and cosine in the model. Transect point and recording day were included as random effects. To keep type I error rate at the nominal level of 5% (Schielzeth & Forstmeier, 2009; Barr et al., 2013) we included all possible random slopes components (sine and cosine of time within both transect point and recording day and pond within recording day) and also the respective correlations between random slopes and intercepts. As an overall test of the fixed effects, we compared the full model with a null model lacking the fixed effects but comprising the same random effects structure as the full model (Forstmeier & Schielzeth, 2011) using a likelihood ratio test (Dobson & Barnett, 2008). We assessed model stability by comparing the estimates derived by a model based on all data with those obtained from models with the levels of random effects excluded one at a time. This revealed the model to be stable. To rule out collinearity we determined Variance Inflation Factors (VIF, Field, 2005) for a standard linear model excluding random effects and interactions. It revealed a VIF of 1.000 for sine and cosine time and pond which means that there was no collinearity issue.

As data were not normally distributed, correlations between indices and aural analysis were calculated using Spearman’s formula. To investigate relationships between acoustic indices and richness and abundance of sounds aurally and visually determined, we performed correlations between acoustic indices (*Ht*, *Hf*, *M*, *AR*, *ACI* and *NP*) and the richness and abundance of sound types. To take into account the effect of the noise on the indices we first estimated the correlation between the acoustic indices (*Ht*, *Hf*, *M*, *AR*, *ACI* and *NP*) and the SNR. Then to control for the correlation between the noise and the indices when investigating the relationship between indices and sound type richness and abundance, we used partial Spearman correlations controlling SNR (Kim, 2012).

All statistical analyses were run with the R statistical controlling for SNR environment (R Core Team, 2015) with the packages ade4 version 1.7-2 (Dray & Dufour, 2007), ppcor version 1.0 (Kim, 2012), lme4 version 1.1-7 (Bates et al., 2014) and car version 2.0-25 (Fox & Weisberg, 2011).

## Results

### Pond acoustic richness

**Figure 1:**
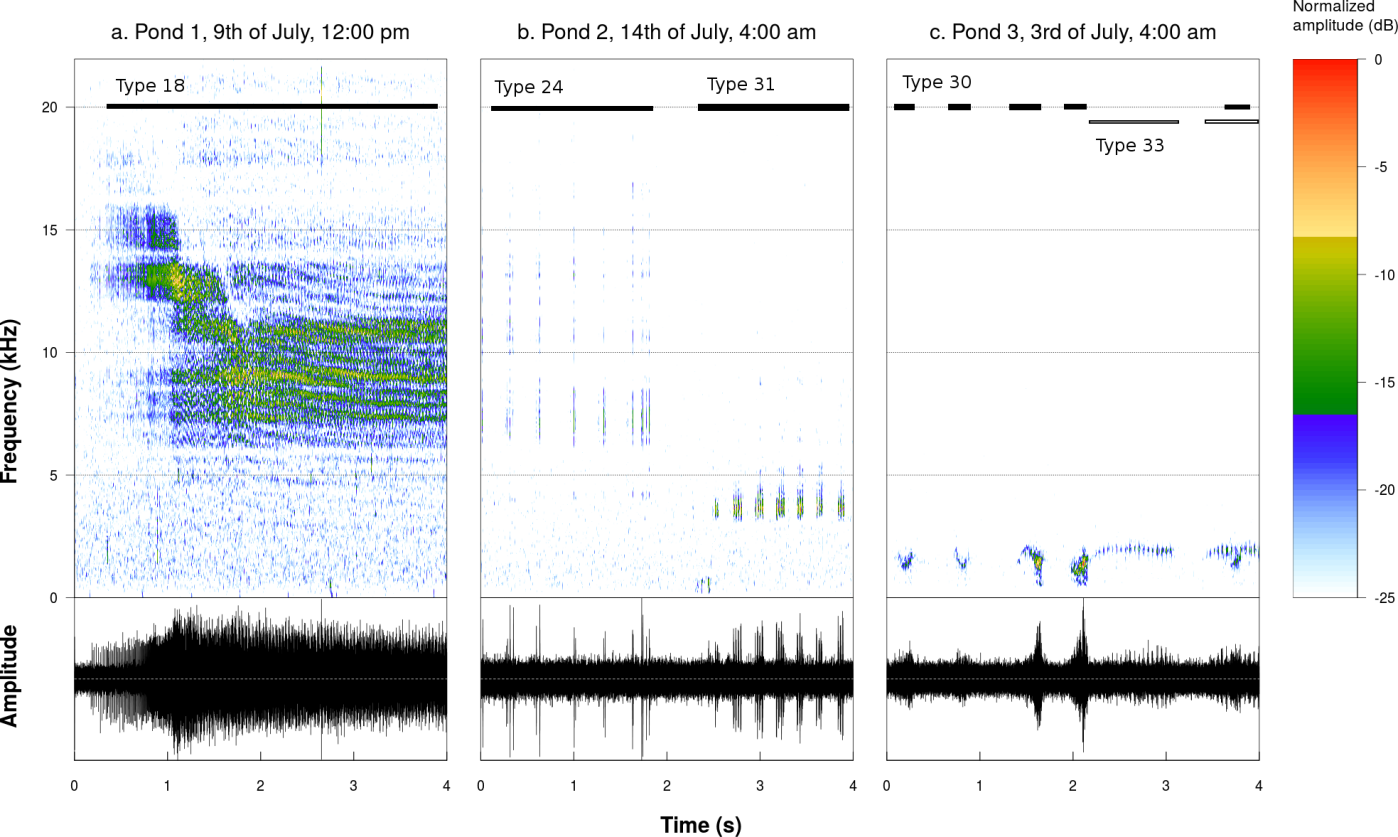
Spectrograms and oscillograms of chosen sound productions illustrating the acoustic diversity found in the studied ponds (Fourier window length: 1,024 samples, frame overlap: 50%, window type: Hanning). (A) Sound type 18 recorded in pond 1 on the 9th of July at 12:00 pm. (B) Sound types 24 and 31 recorded in pond 2 on the 3rd of July at 4:00 am. (C) Sound types 30 and 33 recorded in pond 3 on the 14th of July at 4:00 am. Sound type numbers refer to Supplemental Information.

**Figure 2:**
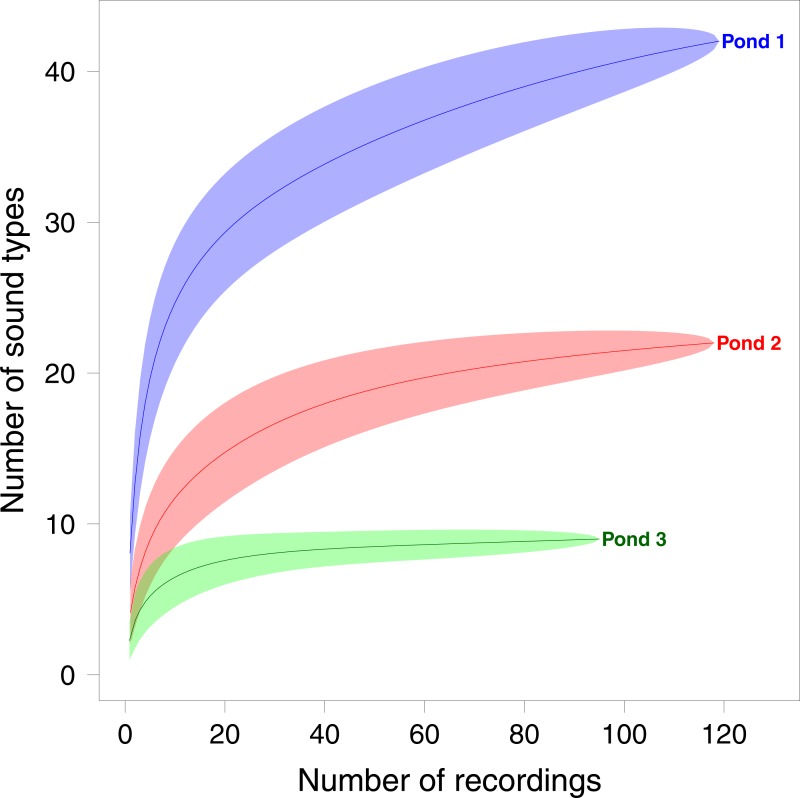
Sample based rarefaction curves of sound types per recording for each pond. Each one minute recording is a sample and the sound types are equivalent to species in the rarefaction process. Shaded area around the curves indicates 95% confidence intervals.

A total of 2,446 sounds were detected and allocated to 48 sound types (see 3 examples in Fig. 1). 48 sound types were identified in pond 1, 22 in pond 2, 9 in pond 3. Pond 1 and pond 2 shared 18 sound types, pond 1 and 3 shared 6 sound types and ponds 2 and 3 shared 7 sound types. The rarefaction curve showed a plateau for both pond 2 and 3 but not for pond 1 (Fig. 2). The plateau is reached at 22 sound types for pond 2 and 9 for pond 3.

**Figure 3:**
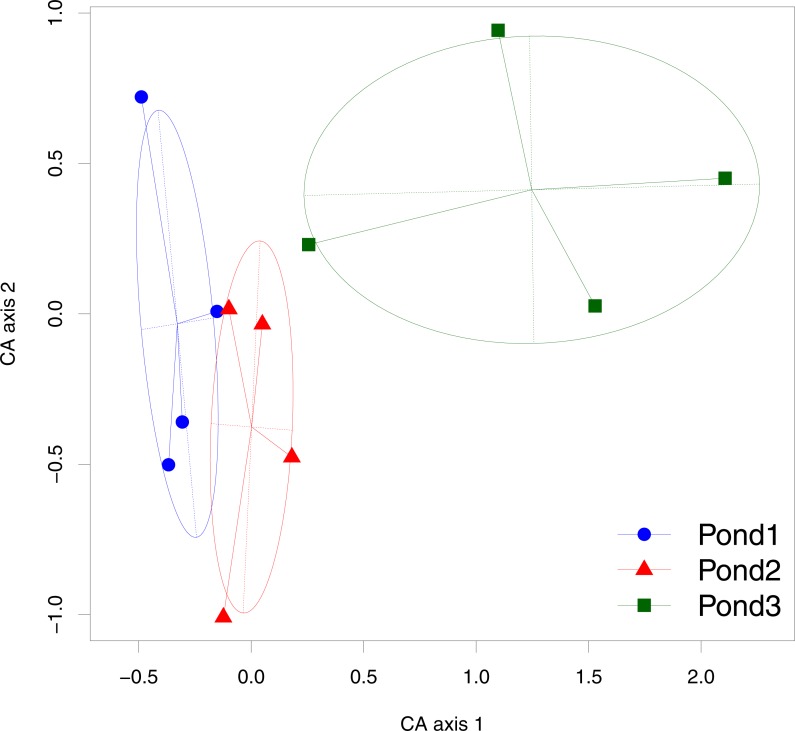
Result of the Correspondence Analysis (CA) with the sound types as variables and the recording points as samples. Each point represents a point of recording in the pond, each ellipse corresponds to 67% of the point dispersion around the centroid for each pond. The axes 1 and 2 explain 26% and 17% of the variance, respectively.

The mean number of sound types found in a recording was 2.2 ± 1.8 (mean ± sd, *n* = 119) for pond 1, 1 ± 1.1 (*n* = 118) for pond 2 and 0.6 ± 0.8 (*n* = 95) for pond 3. The recording points were more similar within ponds than among ponds as shown by the projection of recording sites along the axes 1 and 2 of a CA (Fig. 3). The two first axes explained 43% of the total variance.

**Figure 4:**
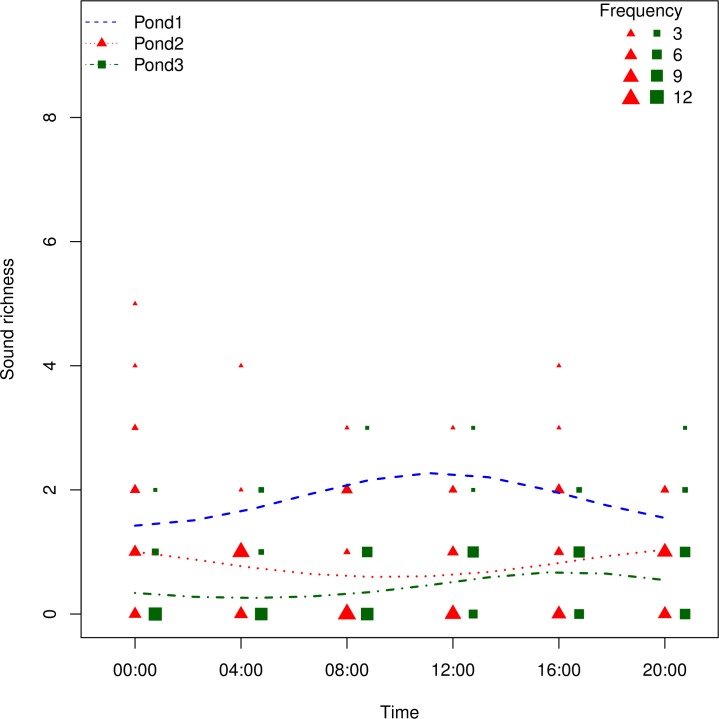
Frequency distribution of sound type richness as a function of time. The size of each point is related to the number of recordings containing the same number of different sound types (total number of recordings *N* = 332). The three dashed lines show the fitted model for each of the three ponds.

There was an impact of pond and time on the richness (likelihood ratio test comparing the full and null model including only the factor pond, *χ*^2^ = 17.269, *df* = 8, *p*-value = 0.027). The daily variation was different from one pond to the other (significant interaction between ponds and time, likelihood ratio test comparing the full model and the model without the interaction, *χ*^2^ = 10.117, *df* = 4, *p*-value = 0.039). Using the predictions of the model, we found that pond 1 had an overall higher number of sound types with its highest sound richness at 11:18, pond 2 had an intermediate sound type richness and its highest sound richness at 20:00 and finally pond 3 had the lowest sound type richness with its highest sound richness at 16:22 (Fig. 4).

### Level of SNR

The values of the signal-to-noise ratio had a mean of 5.38 equivalent to 7.31 dB and a median of 1.12 equivalent to 0.5 dB. These values were variable with a standard deviation of 24.51 equivalent to 14 dB, and a median absolute deviation of 1.67 equivalent to 2 dB. 137 out of 332 recordings (41%) had a SNR lower than 1 equivalent to 0 dB. The SNR levels differed significantly between pond 1 and 2 and pond 1 and 3 but not between pond 2 and 3 (pairwise Wilcoxon test, *p*-values adjusted with the Bonferroni correction: pond 1− pond 2 < 0.0001, pond 1− pond 3 < 0.0001, pond 2− pond 3 = 0.33, *n* = 332). Pond 1 had a significantly higher SNR.

### Correlation with the acoustic indices

Correlations between indices and aural analysis revealed that the indices *Ht* and *Hf* were negatively correlated with the SNR, the sound type richness and the sound type abundance (Table 1). The indices *M*, *ACI* and *NP* were positively correlated with the SNR and both sound type richness and abundance. The index *AR* was negatively correlated with SNR but was not correlated either with sound type richness or abundance (Table 1). The richness and abundance of sound types were correlated with the SNR (respectively 0.80 and 0.84, *p*-values < 0.001). Partial correlations given the SNR revealed that *AR* was positively correlated with both the richness and abundance of sound types. All other indices were not significantly correlated with either abundance or richness given the SNR (Table 2).

**Table 1:** Spearman correlations between acoustic indices (*Ht*, *Hf*, *M*, *AR*, *ACI* and *NP*) and richness and abundance of sound types and SNR.

	*Ht*	*Hf*	*M*	*AR*	*NP*	*ACI*
SNR	−0.61***	−0.41***	0.48***	−0.19*	0.42***	0.55***
Richness	−0.5***	−0.3***	0.44***	−0.04	0.34***	0.49***
Abundance	−0.53***	−0.34***	0.47***	−0.06	0.36***	0.5***

**Notes.**

Stars indicate the significance of the correlation test.

Bonferroni adjusted *p*-value ^∗^ < 0.05, ^∗∗^ < 0.01, ^∗∗∗^ < 0.001.

**Table 2:** Spearman partial correlations between acoustic indices (*Ht*, *Hf*, *M*, *AR*, *ACI* and *NP*) and richness and abundance of sound types given the SNR.

	*Ht*	*Hf*	*M*	*AR*	*NP*	*ACI*
Richness	−0.02	0.06	0.11	0.2**	0	0.1
Abundance	−0.04	0	0.15	0.19**	0.02	0.08

**Notes.**

Stars indicate the significance of the correlation test.

Bonferroni adjusted *p*-value ^∗^ < 0.05, ^∗∗^, *P* < 0.01; ^∗∗∗^, *P* < 0.001.

## Discussion

The acoustic production of terrestrial and marine animals has been thoroughly studied for more than 60 years in bioacoustic studies (Fletcher, 2014). The research presented here an important sound diversity that is now reconsidered under the theoretical frameworks of soundscape ecology (Farina, 2014) and ecoacoustics to tackle ecological questions (Sueur & Farina, 2015). So far, the acoustics of animal species inhabiting freshwater habitats has been largely neglected probably because of the lack of flagship species or emblematic habitats. However, pioneer entomological studies suggested that several aquatic insect species could produce sound (Aiken, 1985) and recent conservation research determined ponds as high concern for biodiversity conservation (Verberk et al., 2006). Here, using a long-term passive acoustic monitoring approach, we (i) identified a total of 48 sound types differing in occurrence between the ponds, (ii) revealed a 24 h cycle of acoustic activity differing among the ponds, and (iii) suggested that the use of *AR* as an index for automatic monitoring was limited by the need for SNR estimation.

Due to the lack of background research just mentioned above and in particular to the lack of an inventory clearly attributing sound types to species names, we were not able to identify the emitting species of the sound types we inventoried. Only a very small portion of the sound types identified were generated by terrestrial animals (4 sound types were identified as birds) but were rather faint. This probably results from the difference in impedance between air and water making it difficult for sound generated in air to transmit through water. The number of sound types, here named sound richness, is therefore probably an overestimation of the number of singing species as we were not able to assess the intra-specific-diversity and some sound types could also result from plant respiration that generates sonorous air bubbles (Felisberto et al., 2015, C Desjonquères, pers. obs., 2014) or from terrestrial animal such as birds.

Even if sound richness could not be directly linked to a number of species, it represents an original facet of biodiversity that can be studied for its own. The three ponds showed different levels and dynamics of sound richness as illustrated with rarefaction curves commonly used to assess sampling effort in biodiversity studies (Gotelli & Colwell, 2001).

The number of recordings appeared sufficient to capture the sound richness in the ponds 2 and 3 as the rarefaction curves showed a plateau. Conversely, the rarefaction curve of sound types did not reach a plateau for pond 1 which had the highest sound richness. This suggests that pond 1 embedded a richer and more dynamic acoustic community with a higher diversity of sound types than in the two other ponds.

The distribution of the sound types among the three ponds was different enough to clearly pull apart the three ponds through a correspondence analysis indicating different acoustic communities. This multivariate analysis also revealed a higher heterogeneity of the points in pond 3 than in pond 1 and 2. The Generalized Linear Mixed model confirmed the differences in sound richness among ponds and revealed as well differences along time, the sound richness of each pond evolving in a different way along the 24 h cycle. All together, these results suggest that the three ponds harbour three different communities in terms of richness, composition, and abundance. These differences are in accordance with the three distinct ecological conditions we deliberately chose (open, semi-closed, closed habitats).

The signal to noise ratio (SNR) measures the ratio of the amplitude of the signal of interest over the amplitude of the surrounding noise. It is difficult to have reference values as it depends on several parameters such as surrounding noise level, amplitude of the source, distance from the receiver to the sources, obstacles between the source and the receiver or physical parameters of the matter in which the wave travels (e.g., humidity, temperature, viscosity). Compared to values in a terrestrial habitats which are usually comprised between 15 and 25 dB (Dabelsteen & Mathevon, 2002), the SNR was rather low here. A high proportion of recordings had a SNR of 0 dB meaning that they only contained signals which are less or as intense as the background noise. It is now rather difficult to identify whether these low SNR values were due to soft signals and/or loud background noise. The acoustic properties of ponds are unfortunately poorly known (Aiken, 1982; Forrest, Miller & Zagar, 1993) compared to marine environments (Buckingham, 1992). These complex and heterogeneous environments may have very peculiar sound propagation patterns impacting the quality of the recordings, in particular the SNR. Further studies should therefore find solutions to increase the SNR such as removing the background noise with lossless filtering techniques.

Acoustic diversity can be estimated through the identification and count of sound types or species-specific songs. Even if very informative, this approach can be very time demanding when handling large sampling covering hours of audio recordings. Recently, acoustic indices have been developed to get a preliminary estimation of the acoustic diversity without sound or species labelling. We therefore tested six alpha acoustic diversity indices. The correlations between the acoustic indices and the aural analysis showed that the entropy based indices *Ht* and *Hf* were negatively correlated with the SNR, the richness and the abundance. This confirms that these metrics are very sensitive to background noise and may function in the reverse way as expected with simulations (Sueur et al., 2008), as it was already pointed out for bird communities (Depraetere et al., 2012; Gasc et al., 2015). Conversely the envelope energy *M*, the number of major peaks of the mean frequency spectrum *NP*, and the Acoustic Complexity Index *ACI* were significantly positively correlated with richness and abundance. Although these three indices have been designed in the aim of circumventing the potential bias induced by the presence of noise and the absence of signals (Depraetere et al., 2012; Gasc et al., 2013), they were here also significantly positively associated with the SNR. Altogether our results show that the SNR could be an important confounding and misleading variable for these five indices and that these indices should be used when recordings have been performed in habitats selected for their low noise level, avoiding flowing water such as streams or waterfalls. The *AR* index was the single index not correlated with neither richness nor abundance and the only index showing a positive and significant correlation with richness and abundance when taking SNR as a control variable in a partial correlation. The index *AR* is therefore a good candidate for revealing acoustic diversities within ponds. One of the major drawback of this index is that its ranking property makes independent studies hard to compare. Moreover our results show that it would be necessary to assess automatically the SNR to use *AR*. To compute the SNR without any manual identification of the signal and noise section within each recording is a technical challenge we could not solve preventing the use of the *AR* index on the complete set of recordings. Development in signal analysis is therefore still required to be able to monitor automatically pond acoustic diversity.

This preliminary study reveals that ponds we sampled were not silent habitats and that each pond revealed a different acoustic diversity. New biodiversity programs should be developed to describe and understand the sound diversity of ponds. Efforts should be achieved through a species-specific approach based on behavioural and systematic sciences to identify the sound sources and, at the same time, through a community approach based on ecological sciences to allow rapid biodiversity assessment. Combining these two research routes should lead to a better knowledge of this still unknown facet of animal diversity.

## Supplemental Information

10.7717/peerj.1393/supp-1 Table S1 Main characteristics of the three ponds sampled

10.7717/peerj.1393/supp-2 Supplemental Information 1 Raw data: acoustic indices and SNR Acoustic indices, SNR, abundance and richness of sound types for each recording.

10.7717/peerj.1393/supp-3 Supplemental Information 2 Raw data: sound types per files Abondance of each sound type per recording.

10.7717/peerj.1393/supp-4 Supplemental Information 3 Noise

10.7717/peerj.1393/supp-5 Supplemental Information 4 Type 04

10.7717/peerj.1393/supp-6 Supplemental Information 5 Type 01

10.7717/peerj.1393/supp-7 Supplemental Information 6 Type 03

10.7717/peerj.1393/supp-8 Supplemental Information 7 Type 08

10.7717/peerj.1393/supp-9 Supplemental Information 8 Type 06

10.7717/peerj.1393/supp-10 Supplemental Information 9 Type 05

10.7717/peerj.1393/supp-11 Supplemental Information 10 Type 11

10.7717/peerj.1393/supp-12 Supplemental Information 11 Type 02

10.7717/peerj.1393/supp-13 Supplemental Information 12 Type 13

10.7717/peerj.1393/supp-14 Supplemental Information 13 Type 14

10.7717/peerj.1393/supp-15 Supplemental Information 14 Type 07

10.7717/peerj.1393/supp-16 Supplemental Information 15 Type 10

10.7717/peerj.1393/supp-17 Supplemental Information 16 Type 12

10.7717/peerj.1393/supp-18 Supplemental Information 17 Type 17

10.7717/peerj.1393/supp-19 Supplemental Information 18 Type 19

10.7717/peerj.1393/supp-20 Supplemental Information 19 Type 16

10.7717/peerj.1393/supp-21 Supplemental Information 20 Type 21

10.7717/peerj.1393/supp-22 Supplemental Information 21 Type 09

10.7717/peerj.1393/supp-23 Supplemental Information 22 Type 22

10.7717/peerj.1393/supp-24 Supplemental Information 23 Type 18

10.7717/peerj.1393/supp-25 Supplemental Information 24 Type 15

10.7717/peerj.1393/supp-26 Supplemental Information 25 Type 23

10.7717/peerj.1393/supp-27 Supplemental Information 26 Type 24

10.7717/peerj.1393/supp-28 Supplemental Information 27 Type 25

10.7717/peerj.1393/supp-29 Supplemental Information 28 Type 26

10.7717/peerj.1393/supp-30 Supplemental Information 29 Type 20

10.7717/peerj.1393/supp-31 Supplemental Information 30 Type 28

10.7717/peerj.1393/supp-32 Supplemental Information 31 Type 31

10.7717/peerj.1393/supp-33 Supplemental Information 32 Type 33

10.7717/peerj.1393/supp-34 Supplemental Information 33 Type 27

10.7717/peerj.1393/supp-35 Supplemental Information 34 Type 34

10.7717/peerj.1393/supp-36 Supplemental Information 35 Type 32

10.7717/peerj.1393/supp-37 Supplemental Information 36 Type 29

10.7717/peerj.1393/supp-38 Supplemental Information 37 Type 30

10.7717/peerj.1393/supp-39 Supplemental Information 38 Type 37

10.7717/peerj.1393/supp-40 Supplemental Information 39 Type 36

10.7717/peerj.1393/supp-41 Supplemental Information 40 Type 39

10.7717/peerj.1393/supp-42 Supplemental Information 41 Type 41

10.7717/peerj.1393/supp-43 Supplemental Information 42 Type 38

10.7717/peerj.1393/supp-44 Supplemental Information 43 Type 44

10.7717/peerj.1393/supp-45 Supplemental Information 44 Type 40

10.7717/peerj.1393/supp-46 Supplemental Information 45 Type 42

10.7717/peerj.1393/supp-47 Supplemental Information 46 Type 43

10.7717/peerj.1393/supp-48 Supplemental Information 47 Type 48

10.7717/peerj.1393/supp-49 Supplemental Information 48 Type 47

10.7717/peerj.1393/supp-50 Supplemental Information 49 Type 46

10.7717/peerj.1393/supp-51 Supplemental Information 50 Type 35

10.7717/peerj.1393/supp-52 Supplemental Information 51 Type 45
