# Supplementary material for: First description of underwater acoustic diversity in three temperate ponds
Source: PeerJ. 2015 Nov 5;3:e1393. doi: 10.7717/peerj.1393 (PMC4647551; doi:10.7717/peerj.1393)
Supplement: Table S1 [file peerj-03-1393-s001.doc]

|  | *Pond 1* | *Pond 2* | *Pond 3* |
| --- | --- | --- | --- |
| Geographical position | 48°34.523’N,  1°53.341’E | 48°40.560’N, 1°55.865’E | 48°40.772’N, 1°55.840’E |
| Type of habitat | closed | open | semi-closed |
| Altitude (m) | 168 | 181 | 177 |
| Depth at 1m from the shore (m) | 0.35 | 0.32 | 0.38 |
| Perimeter (m) | 174 | 85 | 170 |
| Nature of the sediment | Silt and clay | silt | silt |
| Hydrophytes (submerged)* | 0 | 0 | 0.5 |
| Helophytes* | 0 | 0.5 | 0 |
| pH | 6.68 | 7.62 | 6.75 |
| [PO_4_] (mg/L) | 0.25 | 0 | 0.20 |
| [NO_2_] (mg/L) | 0.007 | 0 | 0 |

* Frequency of hygrophytes and helophytes on 10 points equally distributed along the perimeter of the pond (Le Viol et al. 2008)
